# Trends in the Use of Cardiac Imaging for Patients With Heart Failure in Canada

**DOI:** 10.1001/jamanetworkopen.2019.8766

**Published:** 2019-08-09

**Authors:** Juarez R. Braga, Howard Leong-Poi, Valeria E. Rac, Peter C. Austin, Heather J. Ross, Douglas S. Lee

**Affiliations:** 1 ICES, University of Toronto, Toronto, Ontario, Canada; 2 Division of Cardiology, University Health Network, Toronto, Ontario, Canada; 3 Division of Cardiology, St Michael’s Hospital, Toronto, Ontario, Canada; 4 Toronto Health Economics and Technology Assessment Collaborative, Toronto General Hospital Research Institute, University Health Network, Toronto, Ontario, Canada; 5 Institute for Health Policy, Management, and Evaluation, University of Toronto, Toronto, Ontario, Canada; 6 Peter Munk Cardiac Centre, University Health Network, Toronto, Ontario, Canada; 7 Ted Rogers Centre for Heart Research, Toronto, Ontario, Canada; 8 Joint Department of Medical Imaging, University Health Network, Toronto, Ontario, Canada

## Abstract

**Importance:**

Cardiac imaging is a component of the provision of medical care for patients with heart failure that has experienced a broad expansion in past decades. However, there is a paucity of studies examining the patterns of use of cardiac imaging modalities in real-world clinical practice.

**Objectives:**

To investigate temporal trends in the use and costs of cardiac imaging for the examination of patients with heart failure in Canada and to examine the association between the institution of an accreditation program and the use of echocardiography.

**Design, Setting, and Participants:**

A repeated cross-sectional study based on population-based administrative databases in Ontario, Canada, of individuals with heart failure identified using a validated algorithm based on hospital admissions and ambulatory physician claims was conducted between April 1, 2002, and March 31, 2017.

**Main Outcomes and Measures:**

The incidence and prevalence of heart failure and the age- and sex-adjusted rate of use and costs of cardiac imaging, including resting and stress echocardiography, myocardial perfusion scintigraphy, invasive coronary angiography, computed tomography, magnetic resonance imaging, and positron emission tomography.

**Results:**

A total of 882 355 adults (50.1% women; median age, 76 years [interquartile range, 66-83 years]) with prevalent heart failure were identified. The age- and sex-standardized prevalence of heart failure remained stable during the study (2.4% [95% CI, 2.4%-2.4%] in 2002 and 2.0% [95% CI, 2.0%-2.0%] in 2016). There was an increase in the rate of use of resting echocardiography, from 386 tests (95% CI, 373-398) per 1000 patients with heart failure in 2002 to 533 (95% CI, 519-547) per 1000 patients in 2011. Coinciding with the initiation of an accreditation program for echocardiography in 2012, there was an immediate reduction in the rate of use (−59.5 tests per 1000 patients with heart failure; *P* < .001), which was followed by a plateau in subsequent years. At the same time, there was a 10.8% relative reduction in the use of myocardial perfusion scintigraphy and an 11.2% relative reduction in the use of invasive coronary angiography from 2011 to 2016 and the incorporation of newer modalities after they became publicly insured health services.

**Conclusions and Relevance:**

These findings suggest that resting echocardiography remains the most used imaging technique for patients with heart failure, exceeding the use of and the cost spent on other modalities. Stabilization in the use of traditional imaging modalities coincided temporally with the emergence of advanced techniques and provincewide quality improvement policy initiatives.

## Key Points

**Question:**

What is the rate of use and what are the costs of different cardiac imaging modalities used to examine patients with heart failure in Canada?

**Findings:**

In this repeated cross-sectional study of 882 355 participants in Ontario, Canada, cardiac imaging of heart failure was based predominantly on the use of resting echocardiography, myocardial perfusion scintigraphy, and invasive coronary angiography. After 2011, there was a stabilization in the use of traditional modalities and incorporation of cardiac computed tomography and magnetic resonance imaging.

**Meaning:**

Provincewide efforts, such as accreditation programs and the adoption of advanced cardiac imaging techniques, may have been associated with changes in physicians’ ordering patterns of cardiac imaging.

## Teaser

This cross-sectional study investigates temporal trends in the use and costs of cardiac imaging for patients with heart failure in Canada and examines the association between the institution of an accreditation program and the use of echocardiography.

## Introduction

Heart failure (HF) is a major public health problem. In Canada, the direct annual cost associated with the management of HF has been estimated at $2.8 billion, while, in the United States, the total cost was estimated at US$31 billion in 2012 and is projected to increase to $70 billion in 2030.^1,2^ Cardiac imaging is a growing component of the provision of medical care for individuals with HF.^3^ Although echocardiography is still the foundational imaging technique in the investigation of HF,^4^ the armamentarium of diagnostic tools has expanded in recent years. Access to other cardiac imaging modalities is now considered essential because of their utility in identifying underlying causes,^5^ risk stratification, and selection of therapies.^4^

The observed expansion of services has placed greater scrutiny on cardiac imaging.^6^ Although there is an understanding that, the need for cardiac imaging increases as individuals live longer with HF, there have been concerns about the excessive volume of imaging procedures without justification for their use. In Ontario, Canada, cardiac imaging has been an area of interest for policy makers, and several initiatives have been implemented in the past decade to control the use of cardiac imaging, including fee cuts, mandatory prior authorization by an expert panel, and an accreditation program for the provision of echocardiography.^7^

Attesting to the importance of cardiac imaging for HF, several publications have provided advice for the use of cardiac imaging in the investigation of this condition.^4,8^ However, there is a paucity of studies examining the patterns of use of different imaging modalities in real-world clinical practice and whether policy reforms are achieving their goal of controlling the use of cardiac imaging. Therefore, our primary objective was to investigate the temporal trends in the use and costs of cardiac imaging for patients with HF in the context of a system providing universal health care coverage. Our secondary objectives were to examine whether an accreditation program for the provision of echocardiography was associated with temporal changes in the use of this modality.

## Methods

### Data Sources

This population-based study used administrative health care data from Ontario, Canada. A repeated cross-sectional design was used and followed the Strengthening the Reporting of Observational Studies in Epidemiology (STROBE) reporting guidelines for cross-sectional studies.^9^ All residents of Ontario qualify for health care services from a single-payer system. A unique, encoded identifier permitted linkage across administrative databases. Individuals with a diagnosis of HF were identified using the Ontario HF Cohort, a database of all patients with HF, which is created from the Canadian Institute for Health Information’s Discharge Abstract Database (CIHI-DAD; in-hospital outcomes), the Ontario Health Insurance Plan (OHIP; physician claims), and the Registered Persons Database (demographics and vital status). The database defines a diagnosis of HF if a patient has either 1 documented admission with HF in any diagnostic field in the discharge abstract or 1 outpatient claim for HF followed by at least 1 additional outpatient claim within 1 year (the codes used to identify HF are listed in eTable 1 in the Supplement). The date of hospital admission or the first outpatient visit, whichever occurred first, represented the date of diagnosis. This identification method is based on a validated algorithm with 84.8% sensitivity and 97.0% specificity.^10^ The use of data in this project was authorized under section 45 of Ontario’s Personal Health Information Protection Act, a provincial legislation, which does not require review by a research ethics board and waives the need for consent.^11^ Access to data used to conduct this study was granted via ICES, an organisation prescribed under Ontario’s Personal Health Information Protection Act (PHIPA), which is specifically authorized to collect, use, and disclose personal health information, without requiring the approval of a research ethics board, nor the consent of the individuals, in a manner compliant with policies and procedures which have reviewed and approved by the Information and Privacy Commissioner for Ontario, in accordance with section 45(3) of PHIPA.

### Study Cohorts

Having identified all eligible patients with HF, we excluded those younger than 20 years or older than 105 years, non-Ontario residents, or those with an invalid diagnosis date. We assembled 2 study cohorts of (1) prevalent cases of HF and (2) incident cases of HF between April 1, 2002, and March 31, 2017. To create the prevalent cohort, we started the identification of HF claims in April 1, 1997, to ascertain individuals who had received a diagnosis of HF before 2002 and were alive during the study period. A patient was defined as having prevalent HF if he or she was alive at the start of the fiscal year and had a prior diagnosis of HF or met criteria for the diagnosis of HF during that year until the fiscal year in which the patient moved away from the province or died. Thus, a patient could be included in multiple annual cohorts of prevalent cases. Incident cases of HF were defined as those patients who met the criteria for HF and did not have a prior diagnosis of HF after scanning all health records in the previous 5 years. An incident case was included only in the fiscal year during which the patient received the diagnosis.

### Cardiac Imaging Modalities

We examined the use of resting and stress echocardiography, myocardial perfusion scintigraphy (MPS), and invasive coronary angiography (ICA), all of which were referred to as traditional modalities. In addition, we examined the use of coronary computed tomography angiography (CCTA), cardiac magnetic resonance imaging (CMRI), and cardiac positron emission tomography (CPET), all of which were referred to as advanced modalities. Recommendations for cardiac imaging tests for patients with HF according to the American Heart Association and American College of Cardiology guidelines^4^ are listed in eTable 2 in the Supplement. Information regarding receipt of noninvasive testing was obtained from the OHIP database, while use of ICA was identified using the CIHI-DAD. Any diagnostic service with multiple claims on the same day was counted only once to avoid duplicate claims. This procedure has been used previously when examining the use of health care resources based on administrative databases.^12^

### Codes Used to Identify Cardiac Imaging Testing

We used the codes for the professional components of claims in the OHIP database to identify cardiac imaging testing. Codes used to identify resting echocardiography, MPS, CMRI, CPET, CCTA, and stress echocardiography are listed in eTable 3 in the Supplement. Cardiac positron emission tomography became a publicly insured health care service in Ontario starting October 1, 2009, CCTA became a publicly insured health care service starting April 1, 2011, and stress echocardiography became a publicly insured health care service starting September 1, 2011.^13,14,15,16^

### Costs

The analysis was conducted from the perspective of a health care payer. To estimate the costs associated with cardiac imaging over time, we calculated the annual costs for each modality, indexing the costs to 2015 by using the fees from the OHIP reimbursement 2015 Schedule of Benefits.^17^ All costs were reported in Canadian dollars.^18^ To estimate the cost of each modality, we included a mean total cost; we also calculated the mean cost of cardiac imaging per each prevalent case of HF over time.

### Statistical Analysis

A descriptive analysis was performed comparing baseline characteristics between prevalent patients in the fiscal year of 2002 and prevalent patients in the fiscal year of 2016. Continuous variables were expressed as the median and interquartile range (25th and 75th percentiles) and compared with the Kruskal-Wallis test. Categorical variables were expressed as the absolute number and proportion and compared using the χ^2^ statistic.

#### Calculation of Prevalence, Incidence, and Rate of Use of Procedure

For each fiscal year, we calculated the age- and sex-standardized prevalence and incidence of HF and the rate of use of cardiac imaging for patients with prevalent HF. The prevalence rate was reported as a percentage, the incidence rate was reported per 100 000 individuals, and the rate of use was reported per 1000 patients with HF. All rates were directly standardized using the 1991 Canadian population as the reference population and presented with exact 95% CIs calculated using the γ distribution.^19^ To identify significant changes over time, we fit linear regression models with fiscal year as the independent variable and the age- and sex-adjusted rate as the dependent variable. The presence of autocorrelation was examined using the Durbin-Watson test. If first-order autocorrelation was detected, the Prais-Winsten estimator was used for adjustment. As additional analyses, we examined the rate of use of cardiac imaging stratified according to urban or rural residence, as previously described,^20^ and the rate of use of cardiac imaging among the incident cohort.

#### Analysis of Temporal Changes

To examine changes in the rate of use of cardiac imaging before and after the initiation of an accreditation program for the provision of echocardiography, segmented linear regression was used.^21^ The 2012 fiscal year was defined as the change point for the data.

#### Analysis of Time Interval Between Repeated Echocardiograms

To further explore if there was a decrease in the rate of receiving another echocardiogram over time, the Andersen-Gill model was used in the subset of individuals who received at least 1 echocardiogram during the study period.^22^ The Andersen-Gill model is a regression model for the analysis of recurrent events in which an individual can contribute to the risk set with multiple echocardiograms as long as the patient is under observation.^23^ Once a patient received an echocardiogram (described as the previous echocardiogram), the outcome was the time to the next echocardiogram. Once a patient had a next echocardiogram, the preceding echocardiogram became the previous echocardiogram. The model was adjusted for age, sex, and year the patient received the previous echocardiogram. A hazard ratio of less than 1 for the variable of year the patients received the previous echocardiogram would mean that the rate of a repeated echocardiogram decreased with each additional year. Dependence of repeated events within the same individual was accounted for using robust standard errors.^24^ Results were expressed as hazard ratios and 95% CIs. A 2-sided *P* < .05 was considered statistically significant. All analyses were performed using SAS, version 9.4 (SAS Institute Inc).

## Results

### Study Cohorts

The cohort of prevalent cases of HF included 882 355 unique individuals with a median age of 76 years (interquartile range, 66-83 years; 50.1% women), while the cohort of incident cases of HF included 555 603 unique individuals with a median age of 76 years (interquartile range, 66-84 years; 49.8% women). The baseline characteristics of prevalent cases of patients with HF in 2002 and 2016 are presented in eTable 4 in the Supplement.

### Epidemiologic Characteristics of HF

#### Population Prevalence of HF

**Figure 1.**
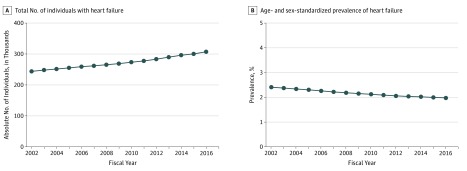
Incidence and Prevalence of Heart Failure, 2002-2016 A, Total number of individuals with a diagnosis of heart failure, 2002-2016. B, Age- and sex-standardized prevalence of heart failure, 2002-2016.

From 2002 to 2016, the number of prevalent cases of HF increased by 25.9%, from 243 882 to 307 023 (Figure 1A). The age- and sex-standardized prevalence of HF remained relatively stable, ranging from 2.4% (95% CI, 2.4%-2.4%) in 2002 to 2.0% (95% CI, 2.0%-2.0%) in 2016 (*P* < .001) (Figure 1B).

#### Incidence of HF

The annual number of incident cases of HF was stable during the study period, ranging from 38 560 in 2002 to 39 754 in 2016 (eTable 5 in the Supplement). The age- and sex-standardized incidence rate decreased from 380 cases (95% CI, 376-384) per 100 000 individuals in 2002 to 256 cases (95% CI, 254-259) per 100 000 individuals in 2016 (*P* < .001).

### Use of Cardiac Imaging

#### Resting Echocardiography

**Figure 2.**
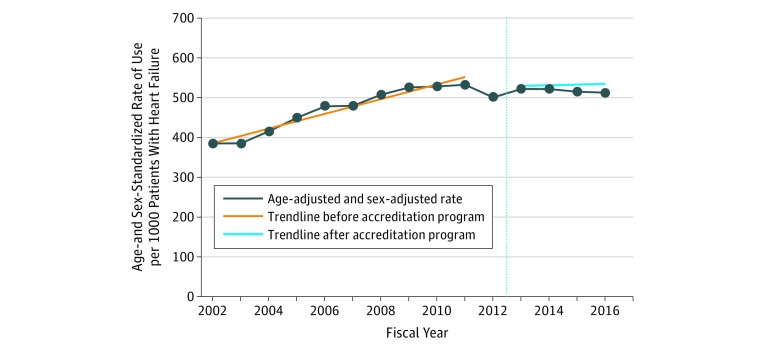
Temporal Changes in the Use of Transthoracic Echocardiography Among Individuals With Heart Failure After the Accreditation Program for the Provision of Echocardiography in Ontario, Canada, 2002-2016 The vertical dotted line represents the fiscal year when the accreditation program was started in Ontario.

The absolute number of resting echocardiograms and other cardiac imaging modalities performed for patients with HF are presented in eTable 6 in the Supplement. The age- and sex-standardized rate of use of resting echocardiography increased 38.1%, from 386 tests (95% CI, 373-398) per 1000 patients with HF in 2002 to 533 tests (95% CI, 519-547) per 1000 patients with HF in 2011 (*P* = .001). Visual inspection of the data revealed that, after reaching a peak in 2011, there was a small reduction in the use of resting echocardiography in 2012, followed by a plateau in subsequent years (Figure 2). Segmented regression analysis revealed that the use of resting echocardiography had a significant annually increasing trend of 18.5 tests per 1000 patients from 2002 to 2011. The start of the accreditation program for resting echocardiography in 2012 was associated with a decrease of 59.5 tests per 1000 patients (*P* < .001) immediately after the start of the program, compared with the level before 2012, and an annual decrease of 16.8 tests per 1000 patients (*P* = .002), compared with the trend before 2012 (eTable 7 in the Supplement). The repeated-events Cox proportional hazards regression analysis revealed that there was a slight decrease in the time for a repeated resting echocardiogram (hazard ratio, 1.033 [95% CI, 1.032-1.034]; *P* < .001) with each increase in year according to the year that the echocardiogram was received.

#### Stress Echocardiography

**Figure 3.**
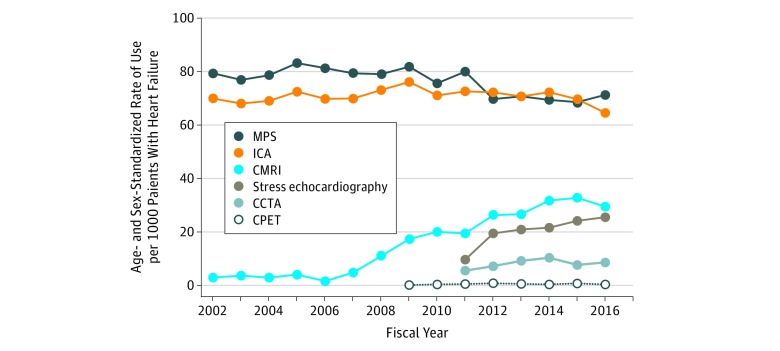
Age- and Sex-Standardized Use of Other Cardiac Imaging Modalities Among Patients With Prevalent Cases of Heart Failure, 2002-2016 CCTA indicates coronary computed tomography angiography; CMRI, cardiac magnetic resonance imaging; CPET, cardiac positron emission tomography; ICA, invasive coronary angiography; and MPS, myocardial perfusion scintigraphy.

The age- and sex-standardized rate of use of stress echocardiography increased from 10 tests (95% CI, 8-11) per 1000 patients with HF in 2011 to 25 tests (95% CI, 23-28) per 1000 patients with HF in 2016 (*P* = .03) (Figure 3). Stress echocardiograms represented less than 5% of the total number of echocardiograms obtained in 2016.

#### Myocardial Perfusion Scintigraphy

The age- and sex-standardized rate of use of MPS remained stable from 2002 to 2011, ranging from 79 tests (95% CI, 75-84) per 1000 patients with HF to 80 tests (95% CI, 76-84) per 1000 patients with HF. Starting in 2012, there was a decrease in the rate of use of MPS, decreasing to 70 tests (95% CI, 66-73) per 1000 patients with HF and remaining stable thereafter (*P* = .008) (Figure 3).

#### Invasive Coronary Angiography

The age- and sex-standardized rate of use of ICA had been stable during the observation period, fluctuating at around 70 tests per 1000 patients with HF. However, the rate decreased in the last 3 years of the study to 64 tests (95% CI, 61-68) per 1000 individuals with HF (*P* = .75) (Figure 3).

#### Advanced Cardiac Imaging

The age- and sex-standardized rate of use of CCTA per 1000 patients with HF ranged from 6 tests (95% CI, 4-7) in 2011 to 9 tests (95% CI, 7-10) in 2016 (*P* = .33). The age- and sex-standardized rate of use of CMRI per 1000 patients with HF increased from 3 tests (95% CI, 2-5) in 2002 to 30 tests (95% CI, 26-33) in 2016 (*P* < .001). The age- and sex-standardized rate of use of CPET per 1000 patients with HF ranged from 0.1 tests (95% CI, 0.08-0.2) in 2009 to 0.4 tests (95% CI, 0.2-0.5) in 2016 (*P* = .39) (Figure 3). With the availability of advanced cardiac imaging starting in 2009, there was a decrease in the ratio of traditional imaging procedures to advanced imaging procedures performed over time (eFigure 1 in the Supplement).

#### Use of Cardiac Imaging According to Rurality and Among the Incident HF Cohort

The rate of use of cardiac imaging, especially echocardiography and ICA, among patients in the incident HF cohort was higher in the year in which patients received a diagnosis of HF compared with the prevalent cohort. However, temporal trends in the incident HF cohort were similar to those observed among patients in the prevalent HF cohort (eFigure 2 and eFigure 3 in the Supplement). When examining the use of cardiac imaging according to urban or rural residence, we found that individuals with HF living in rural areas had lower rates of use of resting and stress echocardiography and CMRI, but their rates of use of other imaging tests were similar irrespective of geography, particularly in recent years of study (eFigures 4-6 in the Supplement).

#### Costs

**Figure 4.**
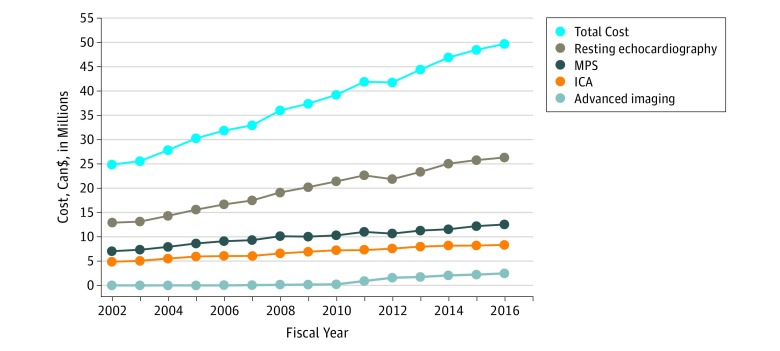
Costs Associated With Cardiac Imaging in the Investigation of Patients With Heart Failure, 2002-2016 ICA indicates invasive coronary angiography; and MPS, myocardial perfusion scintigraphy.

**Figure 5.**
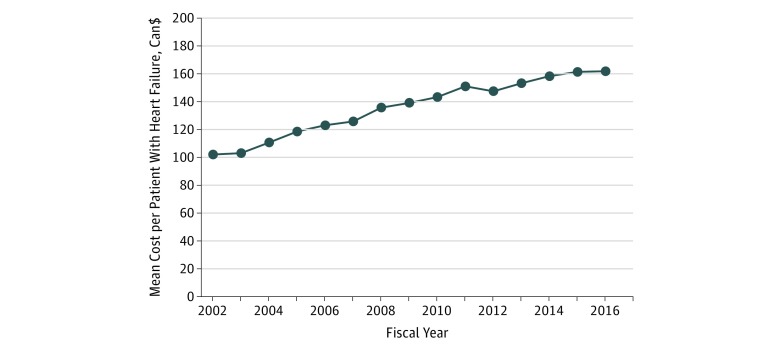
Costs per Capita Associated With Cardiac Imaging in the Investigation of Patients With Heart Failure, 2002-2016

Annual expenditures for cardiac imaging in the investigation of HF increased nearly 2-fold, from Can$24.8 million (US $18.9 million) in 2002 to Can$49.6 million (US $37.8 million) in 2016. Resting echocardiography was responsible for approximately 53% of the total amount spent on cardiac imaging in 2016. The second and third modalities that incurred the highest costs were MPS (25% of all costs) and ICA (17% of all costs). Advanced modalities (CCTA, CMRI, and CPET) were responsible for 5% of all expenses from cardiac imaging in 2016 (Figure 4). The mean cost of cardiac imaging per patient with HF increased from Can$102 (US $78) to Can$162 (US $123) in 2016. However, the mean cost stabilized in the last 2 years of the observed period (Figure 5).

## Discussion

This study examined the use of cardiac imaging for individuals with a diagnosis of HF during a 15-year period in the largest province in Canada. Our data revealed 2 complementary perspectives that need to be dissected to understand the role played by those resources in daily practice. From a clinical perspective, based on the analysis of adjusted rates, we observed that regardless of the potential advantages of advanced imaging modalities, the investigation of HF is still based on a triad of traditional modalities that includes resting echocardiography, MPS, and ICA. Among those traditional modalities, resting echocardiography remains the most used test, by far exceeding that of any other imaging technique.

Furthermore, we observed some trends in our data: the use of resting echocardiography showed rapid increase between 2002 and 2011, which was disproportional to the occurrence of HF in the population. Although the incidence rate of HF has decreased significantly and the prevalence has remained relatively stable, the adjusted use of resting echocardiography increased by 38.1% during the same period. However, after 2011, we observed that the use of resting echocardiography leveled off to an approximately 0% growth, while the use of MPS showed a decrease. Those temporal trends were virtually identical among the incident cohort. Meanwhile, while individuals with HF living in rural areas had lower rates of resting and stress echocardiography and CMRI compared with individuals with HF living in urban areas, the trends in other imaging modalities were similar irrespective of region residence.

Another perspective offered by our study was the health care system point of view. We observed that the number of examinations and the costs for all modalities have shown an increase over time. Our data demonstrated that resting echocardiography was responsible for approximately 53% of all expenditures from cardiac imaging in 2016. Meanwhile, advanced modalities were responsible for only 5% of the total costs. The higher use of resting echocardiography compared with all the other modalities may be because echocardiograms are noninvasive and because there is a perception among health care professionals that the price per unit is low compared with other imaging techniques.^25^ Meanwhile, scanners for advanced technologies are restricted to major centers and are not available for the average patient with HF in the province.^26^ Among the advanced technologies, CPET had the lowest use. Cardiac positron emission tomography is distinct in comparison with other modalities because it requires mandatory prior authorization by a panel composed of radiologists and cardiologists to be insured under OHIP.^27^

In Ontario, several initiatives that could explain the observed trends have been considered and implemented to control the use of cardiac imaging. For instance, in 2012, the provincial government proposed a plan to reduce the physician fee for resting echocardiography by 50% in cases of self-referral. Although this plan was never implemented, it started a discussion about the appropriateness of testing.^28,29^ Also, in 2012, an agency responsible for regulating cardiac care in the province, CorHealth, published standards for the provision of echocardiography and created an accreditation program for echocardiography readers, technicians, and laboratories.^7^ These standards indicate that accredited echocardiography laboratories should ensure that studies meet appropriate use criteria, based on the echocardiography guidelines of the American College of Cardiology, American Heart Association, and American Society of Echocardiography.^30^ A finding of our study was that this accreditation program was followed by an immediate reduction in the use of echocardiography and later by a stabilization in the use of this modality. However, the analysis of repeated examinations demonstrated that there was a decrease in the time to another echocardiography, but the magnitude of change was small.

The pressure to restrain unnecessary testing comes also from physicians who have promulgated appropriate use criteria and the Choosing Wisely Canada Campaign to guide and educate health care professionals and patients about the overuse of diagnostic imaging.^8,31,32,33,34^ Besides those organized efforts, the stabilization and reduction in the use of traditional imaging modalities coincided temporally with the start in coverage under OHIP of advanced imaging modalities. The adoption of new technologies is another factor that could explain the observed temporal changes.

### Implications for Policy and Future Research

The observed trends may guide policy makers as they develop future imaging-related policies. First, CPET was the modality with the lowest use. Although this finding may be related to the few indications that are insured and the lack of province-wide capacity to perform CPET, it is also possible that it may be the consequence of the need for mandatory prior authorization. The disadvantages of this requirement are the negative effects associated with the timeliness of diagnosis, which may be harmful to patients. Second, any research that aims to examine the consequences of policies implemented to restrain the increase in use of a specific modality should consider the overall picture for the management of the condition under investigation. In the scenario of HF, the isolated analysis of resting echocardiography could lead to the notion that quality improvement initiatives are controlling the use of that modality, while in fact one cannot conclusively rule out that the observed trends may be the consequence of substituting traditional modalities for advanced techniques, as suggested by the ratio of traditional to advanced modalities over time. Consequently, the net effect may not necessarily be a reduction in the number of tests and costs.

### Limitations

Our study should be interpreted in the context of its limitations. First, OHIP does not capture a small proportion (<5%) of outpatient physician services owing to the existence of alternate payment plans. It also does not identify some of the cardiac tests performed in inpatient settings because the costs may be absorbed by hospital global budgets and the claims not submitted to OHIP.^35^ Despite these limitations, this study provides the best population estimates associated with the use of cardiac imaging for patients with HF in daily practice. Second, there are several potential reasons that could explain the stabilization and decrease in the use of resting echocardiography, MPS, and ICA starting in 2011. Our study did not allow us to separate the association of each individual initiative with the observed trends. Third, economic effects were determined by the amounts billed for services provided. However, the economic effect of a technology on health care costs may be more complicated by affecting the use of other services either by offsetting savings or inducing costs that were not quantified.

## Conclusions

The results of this study suggest that investigation of HF is still based on the use of resting echocardiography, MPS, and ICA. Resting echocardiography remains the most used technique, exceeding the rate of use and total spending on any other modality, with a rapid increase until 2011. After 2011, there was a decrease in the use of echocardiography, ICA, and MPS that coincided with the emergence of advanced techniques and provincewide quality improvement initiatives to control the use of cardiac imaging.

## Notes

Supplement. **eTable 1.** Codes Used to Identify a Diagnosis of Heart Failure Using Administrative Databases **eTable 2.** Recommendations for the Use of Cardiac Imaging Modalities Among Patients With Heart Failure According to the 2013 American Guidelines **eTable 3.** Codes Used to Identify Modalities of Cardiac Imaging Using Administrative Databases **eTable 4.** Comparison of Baseline Characteristics Between Prevalent Patients in the Fiscal Years of 2002 and 2016 **eTable 5.** Population of Ontario and Total and New Cases of Heart Failure per Fiscal Year, 2002-2016 **eTable 6.** Number of Exams Performed Among Individuals With HF in Ontario per Fiscal Year, 2002-2016 **eTable 7.** Results for the Segmented Linear Regression Analysis Examining the Age- Sex- Standardized Utilization Rate of Rest Echocardiography Before and After 2012 **eFigure 1.** Traditional:Advanced Cardiac Imaging Modalities Ratio, 2009-2016 **eFigure 2.** Age- and Sex-Standardized Utilization of Rest Echocardiography Among Individuals With Incident Heart Failure, 2002-2016 **eFigure 3.** Age- and Sex-Standardized Utilization of Other Cardiac Imaging Modalities Among Individuals With Incident Heart Failure, 2002-2016 **eFigure 4.** Age- and Sex-Standardized Utilization of Rest Echocardiography Among Prevalent Cases of Heart Failure According to the Place of Living, 2002-2016 **eFigure 5.** Age- and Sex-Standardized Utilization of Traditional Cardiac Imaging Modalities Among Prevalent Cases of Heart Failure According to the Place of Living, 2002-2016 **eFigure 6.** Age- and Sex-Standardized Utilization of Advanced Cardiac Imaging Modalities Among Prevalent Cases of Heart Failure According to the Place of Living, 2002-2016
