# Supplementary material for: Trends in the Use of Cardiac Imaging for Patients With Heart Failure in Canada
Source: JAMA Netw Open. 2019 Aug 9;2(8):e198766. doi: 10.1001/jamanetworkopen.2019.8766 (PMC6692835; doi:10.1001/jamanetworkopen.2019.8766)
Supplement: Supplement. — eTable 1. Codes Used to Identify a Diagnosis of Heart Failure Using Administrative Databases eTable 2. Recommendations for the Use of Cardiac Imaging Modalities Among Patients With Heart Failure According to the 2013 American Guidelines eTable 3. Codes Used to Identify Modalities of Cardiac Imaging Using Administrative Databases eTable 4. Comparison of Baseline Characteristics Between Prevalent Patients in the Fiscal Years of 2002 and 2016 eTable 5. Population of Ontario and Total and New Cases of Heart Failure per Fiscal Year, 2002-2016 eTable 6. Number of Exams Performed Among Individuals With HF in Ontario per Fiscal Year, 2002-2016 eTable 7. Results for the Segmented Linear Regression Analysis Examining the Age- Sex- Standardized Utilization Rate of Rest Echocardiography Before and After 2012 eFigure 1. Traditional:Advanced Cardiac Imaging Modalities Ratio, 2009-2016 eFigure 2. Age- and Sex-Standardized Utilization of Rest Echocardiography Among Individuals With Incident Heart Failure, 2002-2016 eFigure 3. Age- and Sex-Standardized Utilization of Other Cardiac Imaging Modalities Among Individuals With Incident Heart Failure, 2002-2016 eFigure 4. Age- and Sex-Standardized Utilization of Rest Echocardiography Among Prevalent Cases of Heart Failure According to the Place of Living, 2002-2016 eFigure 5. Age- and Sex-Standardized Utilization of Traditional Cardiac Imaging Modalities Among Prevalent Cases of Heart Failure According to the Place of Living, 2002-2016 eFigure 6. Age- and Sex-Standardized Utilization of Advanced Cardiac Imaging Modalities Among Prevalent Cases of Heart Failure According to the Place of Living, 2002-2016 [file jamanetwopen-2-e198766-s001.pdf]

Supplementary Online Content

Braga JR, Leong-Poi H, Rac VE, Austin PC, Ross HJ, Lee DS. Trends in the use of cardiac imaging for patients with heart failure in Canada. *JAMA Netw Open.* 2019;2(8):e198766. doi:10.1001/jamanetworkopen.2019.8766

eTable 1. Codes Used to Identify a Diagnosis of Heart Failure Using Administrative Databases
eTable 2. Recommendations for the Use of Cardiac Imaging Modalities Among Patients With Heart Failure According to the 2013 American Guidelines
eTable 3. Codes Used to Identify Modalities of Cardiac Imaging Using Administrative Databases
eTable 4. Comparison of Baseline Characteristics Between Prevalent Patients in the Fiscal Years of 2002 and 2016
eTable 5. Population of Ontario and Total and New Cases of Heart Failure per Fiscal Year, 2002-2016
eTable 6. Number of Exams Performed Among Individuals With HF in Ontario per Fiscal Year, 2002-2016
eTable 7. Results for the Segmented Linear Regression Analysis Examining the Age-Sex- Standardized Utilization Rate of Rest Echocardiography Before and After 2012
eFigure 1. Traditional:Advanced Cardiac Imaging Modalities Ratio, 2009-2016
eFigure 2. Age- and Sex-Standardized Utilization of Rest Echocardiography Among Individuals With Incident Heart Failure, 2002-2016
eFigure 3. Age- and Sex-Standardized Utilization of Other Cardiac Imaging Modalities Among Individuals With Incident Heart Failure, 2002-2016
eFigure 4. Age- and Sex-Standardized Utilization of Rest Echocardiography Among Prevalent Cases of Heart Failure According to the Place of Living, 2002-2016
eFigure 5. Age- and Sex-Standardized Utilization of Traditional Cardiac Imaging Modalities Among Prevalent Cases of Heart Failure According to the Place of Living, 2002-2016
eFigure 6. Age- and Sex-Standardized Utilization of Advanced Cardiac Imaging Modalities Among Prevalent Cases of Heart Failure According to the Place of Living, 2002-2016

This supplementary material has been provided by the authors to give readers additional information about their work.

© 2019 Braga JR et al. *JAMA Network Open.*

eTable 1. Codes Used to Identify a Diagnosis of Heart Failure Using Administrative Databases

Condition	Codes
**Heart failure**	ICD-9-CM codes 428, 414.8, 422, 425, 429, 402.9 plus 428, and 404.9 plus 428 and ICD-10CA codes I50, I25.5, I40, I41, I42, I43, I11 plus I50, and I13 plus I50

ICD-9-CM: International Classification of Diseases Ninth Revision Clinical Modification; ICD-10-CA: International Classification of Diseases Tenth Revision Canadian enhanced version.

© 2019 Braga JR et al. *JAMA Network Open.*

eTable 2. Recommendations for the use of cardiac imaging modalities among patients with heart failure according to the 2013 American guidelines

Level	Class	Recommendation
I	C	An echocardiogram should be performed during initial evaluation of patients presenting with HF to assess ventricular function, size, wall thickness, wall motion, and valve function.
	C	Repeat measurement of LVEF and measurement of structural remodeling are useful to provide information in patients with HF who have had a significant change in clinical status; who have experienced or recovered from a clinical event; or who have received treatment that might have had a significant effect on cardiac function; or who may be candidates for device therapy.
IIa	B	Viability assessment is reasonable in select situations when planning revascularization in HF patients with CAD.
	B	CMRI is reasonable when assessing myocardial infiltrative processes or scar burden.
	C	Non-invasive imaging to detect myocardial ischemia and viability is reasonable in patients presenting with *de novo* HF who have known CAD and no angina unless the patient is not eligible for revascularization of any kind.
	C	Radionuclide ventriculography or CMR can be useful to assess LVEF and volume when echocardiography is inadequate.
	C	When ischemia may be contributing to HF, coronary arteriography is reasonable for patients eligible for revascularization.

CAD: coronary artery disease; CMRI: cardiac resonance imaging; HF: heart failure; LVEF: left ventricular ejection fraction.
Source: Yancy *et al.* 2013 ACCF/AHA guideline for the management of heart failure.

© 2019 Braga JR et al. *JAMA Network Open.*

eTable 3. Codes Used to Identify Modalities of Cardiac Imaging Using Administrative Databases

Modality	Codes
**Rest echocardiography**	G561, G562, G567, G568, G571, G572, and G575[a]
**Myocardial perfusion scintigraphy**	J607, J608, J609, J666, J807, J808, J809, and J866[b]
**Cardiac magnetic resonance imaging**	Combination of code X441 for thorax MRI and code X486 for cardiac gating[c]
**Cardiac positron emission tomography**	J707, J708
**Coronary computed tomography angiography**	X235
**Stress echocardiography**	G583 and G584
**Invasive coronary angiography**	CCP codes 489.4 to 489.8, 499.6 and 499.7 and CCI code 3.IP.10

CCP: Canadian Classification of Diagnostic, Therapeutic, and Surgical Procedures; CCI: Canadian Classification of Health Interventions

a: Codes for rest echocardiography refer to all cardiac ultrasound imaging techniques, including M-mode, 2- and 3-dimensional imaging, and color Doppler.

b: Myocardial perfusion scintigraphy included exercise or pharmacological myocardial perfusion imaging with or without single photon emission computer tomography using either sestamibi or thallium as its radiotracer). Given that perfusion imaging tests may be conducted over 1 or more consecutive days, we applied a 2-day window on either side of the date of a scintigraphy claim to avoid duplicate counting.

c: This combination has been previously validated to identify receipt of cardiac magnetic resonance imaging. Roifman et al. Validation of billing code combinations to identify cardiac magnetic resonance imaging scans in Ontario, Canada. BMJ Open, In press.

© 2019 Braga JR et al. *JAMA Network Open.*

eTable 4. Comparison of Baseline Characteristics Between Prevalent Patients in the Fiscal Years of 2002 and 2016

Variable	2002 (N=243,882)	2016 (N=307,023)	P-value
**Age, yrs, median (25th-75th)**	76 (67-83)	76 (66-85)	<.001
**Female sex, n (%)**	124,624 (51.1)	146,757 (47.8)	<.001
**Rural residence, n (%)**	42,680 (17.5)	40,527 (13.2)	<.001
**Medical history, n (%)**			
Atrial fibrillation/flutter	51,460 (21.1)	68,159 (22.2)	<.001
Cancer	20,974 (8.6)	27,325 (8.9)	<.001
Chronic kidney disease	24,632 (10.1)	62,326 (20.3)	<.001
COPD	46,338 (19.0)	44,211 (14.4)	<.001
Dementia	17,803 (7.3)	31,009 (10.1)	<.001
Depression	16,096 (6.6)	17,807 (5.8)	<.001
Diabetes	64,629 (26.5)	104,080 (33.9)	<.001
Hypertension	135,355 (55.5)	168,862 (55.0)	<.001
Liver cirrhosis	3,171 (1.3)	6,755 (2.2)	<.001
Previous AMI	37,556 (15.4)	30,702 (10.0)	<.001
Previous HF hospitalization	99,016 (40.6)	91,800 (29.9)	<.001
Peripheral vascular disease	24,632 (10.1)	14,737 (4.8)	<.001
Stroke	35,363 (14.5)	29,474 (9.6)	<.001

AMI: acute myocardial infarction; COPD: chronic obstructive pulmonary disease; HF: heart failure; N: number; yrs: years of age.

© 2019 Braga JR et al. *JAMA Network Open.*

eTable 5. Population of Ontario and Total and New Cases of Heart Failure per Fiscal Year, 2002-2016

Fiscal Year	Ontario population*	Total cases of HF	New cases of HF
**2002**	8,961,237	243,882	38,560
**2003**	9,112,530	248,036	36,765
**2004**	9,258,879	251,424	36,082
**2005**	9,398,756	255,176	35,994
**2006**	9,533,217	259,007	35,499
**2007**	9,646,382	261,772	34,862
**2008**	9,764,732	265,253	36,348
**2009**	9,884,030	268,812	35,467
**2010**	10,026,231	273,538	35,821
**2011**	10,160,334	277,491	35,700
**2012**	10,328,123	283,240	36,731
**2013**	10,485,643	289,699	38,513
**2014**	10,628,572	296,176	40,668
**2015**	10,753,893	300,276	38,839
**2016**	10,929,424	307,023	39,754

* Older than 20 years of age.
HF: heart failure.

© 2019 Braga JR et al. *JAMA Network Open.*

eTable 6. Number of Exams Performed Among Individuals With HF in Ontario per Fiscal Year, 2002-2016

Fiscal Year	Rest echo	MPS	ICA	Stress echo	CCTA	CMRI	CPET
**2002**	63,362	15,176	11,976	-	-	93	-
**2003**	64,403	15,815	12,417	-	-	76	-
**2004**	70,212	17,121	13,565	-	-	104	-
**2005**	76,516	18,634	14,612	-	-	127	-
**2006**	81,772	19,639	14,797	-	-	125	-
**2007**	85,692	20,100	14,870	-	-	236	-
**2008**	93,772	21,854	16,164	-	-	505	-
**2009**	99,102	21,647	17,003	-	-	651	35
**2010**	104,970	22,201	17,763	-	-	798	70
**2011**	111,055	23,798	17,834	1804	681	923	102
**2012**	107,222	23,024	18,622	3708	885	1159	146
**2013**	114,525	24,333	19,590	3916	1187	1277	175
**2014**	122,817	24,918	20,076	4617	1395	1601	160
**2015**	126,385	26,329	20,133	4938	1631	1770	125
**2016**	129,009	27,038	20,436	5555	1760	1862	129

The claims code for CPET were introduced in 2009, while the codes for CCTA and stress echocardiography were introduced in 2011.
-: empty cell; CCTA: coronary computed tomography angiography; CMRI: cardiac magnetic resonance imaging; CPET: cardiac positron emission tomography; ICA: invasive coronary angiography; MPS: myocardial perfusion scintigraphy.

© 2019 Braga JR et al. *JAMA Network Open.*

eTable 7. Results for the Segmented Linear Regression Analysis Examining the Age- Sex-Standardized Utilization Rate of Rest Echocardiography Before and After 2012

Parameter	Coefficient	Standard error	t value	*P*-value
Intercept	367.5	8.5895	42.78	<.001
Pre-intervention slope	18.5	1.4	13.36	<.001
Change in intercept	-59.5	13.0	-4.58	<.001
Change in slope	-16.8	4.2	-3.99	0.002

© 2019 Braga JR et al. *JAMA Network Open.*

eFigure 1. Traditional:Advanced Cardiac Imaging Modalities Ratio, 2009-2016

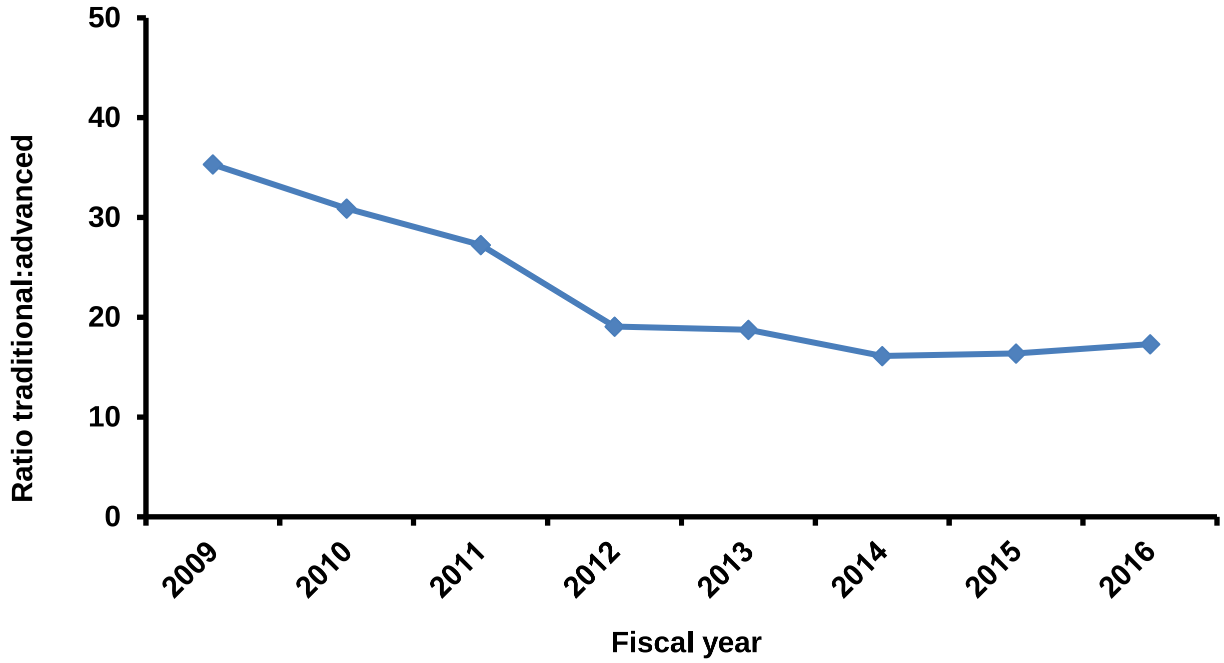

© 2019 Braga JR et al. *JAMA Network Open.*

eFigure 2. Age- and Sex-Standardized Utilization of Rest Echocardiography Among Individuals With Incident Heart Failure, 2002-2016

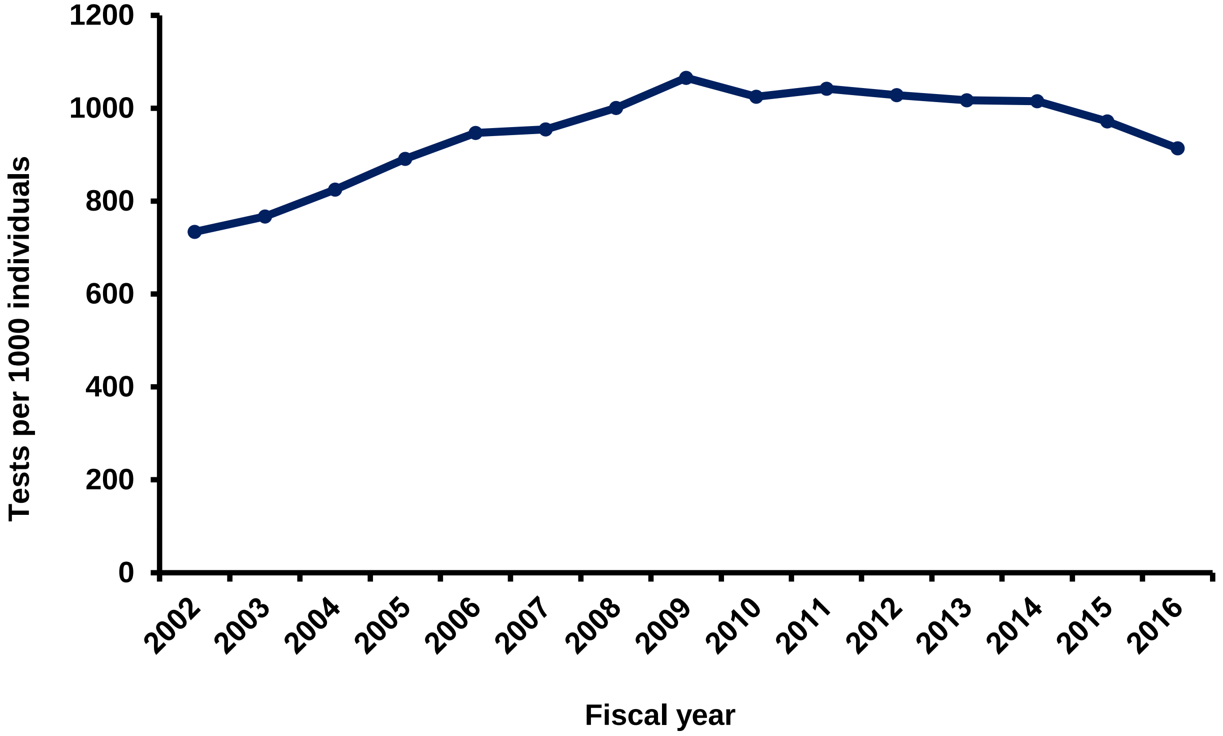

© 2019 Braga JR et al. *JAMA Network Open.*

eFigure 3. Age- and Sex-Standardized Utilization of Other Cardiac Imaging Modalities Among Individuals With Incident Heart Failure, 2002-2016

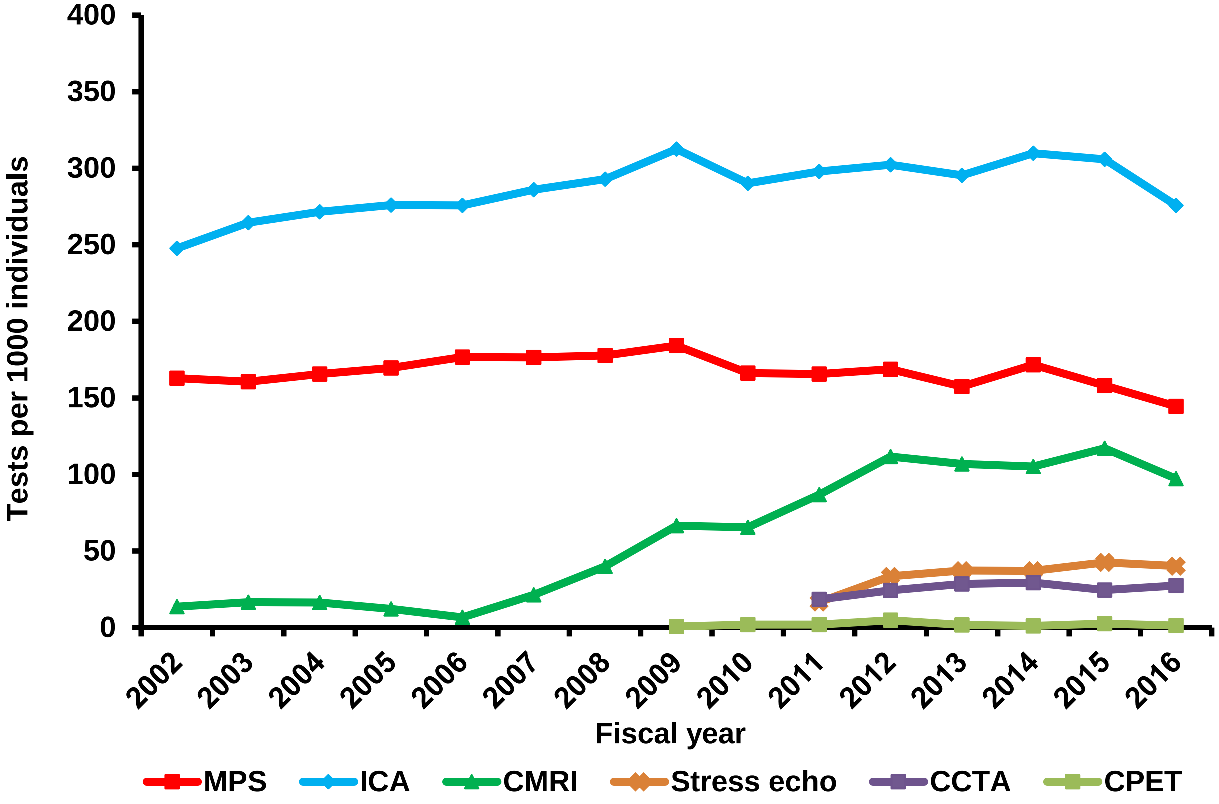

© 2019 Braga JR et al. *JAMA Network Open.*

eFigure 4. Age- and Sex-Standardized Utilization of Rest Echocardiography Among Prevalent Cases of Heart Failure According to the Place of Living, 2002-2016

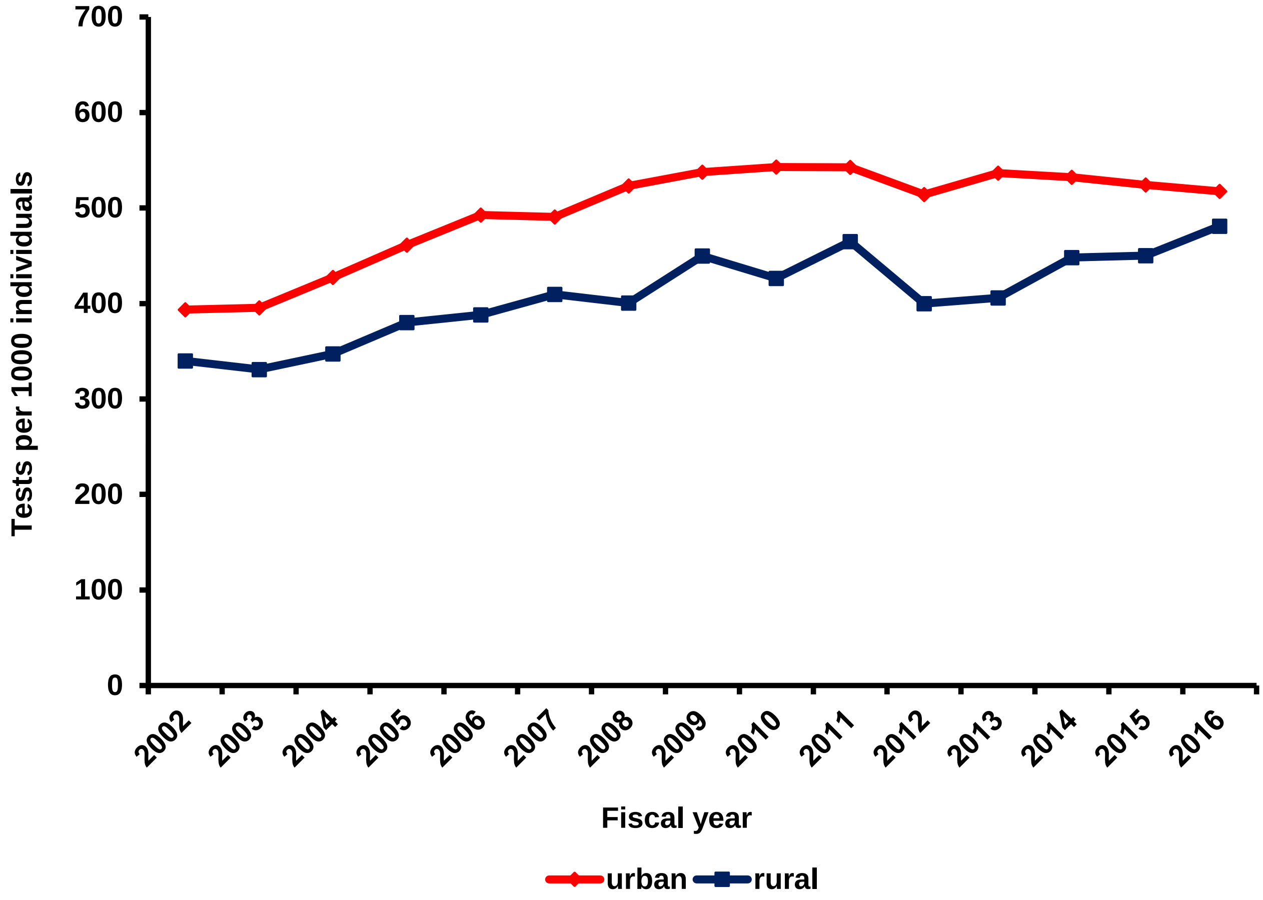

© 2019 Braga JR et al. *JAMA Network Open.*

eFigure 5. Age- and Sex-Standardized Utilization of Traditional Cardiac Imaging Modalities Among Prevalent Cases of Heart Failure According to the Place of Living, 2002-2016

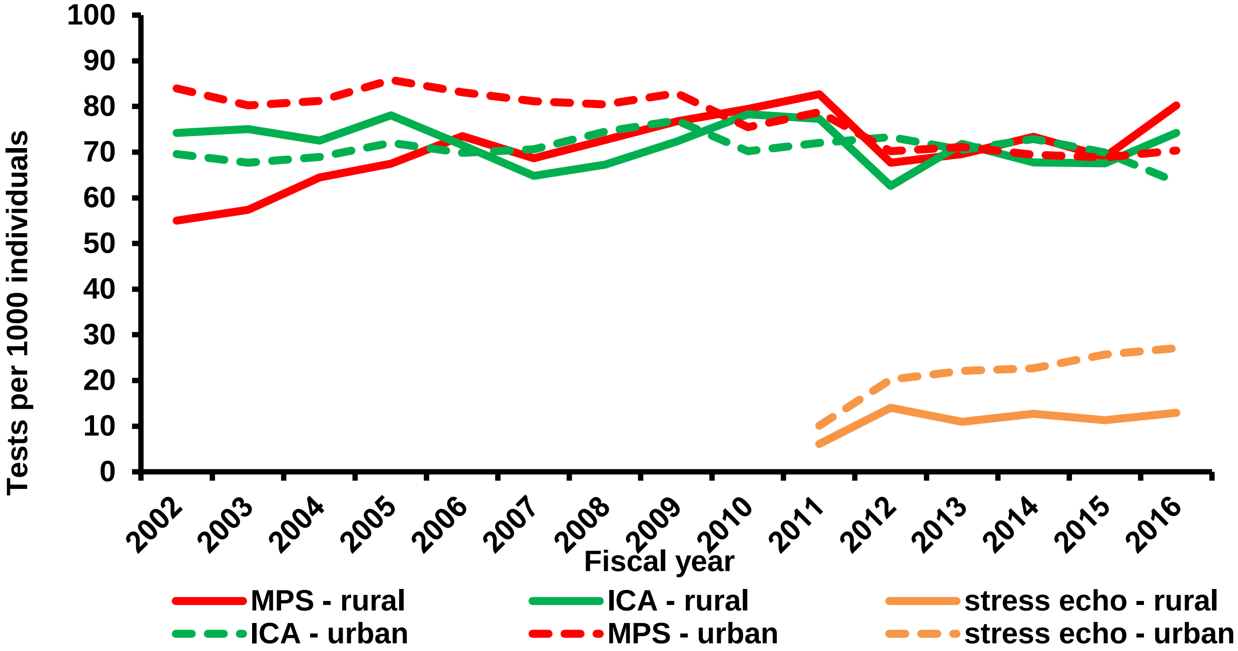

© 2019 Braga JR et al. *JAMA Network Open.*

eFigure 6. Age- and Sex-Standardized Utilization of Advanced Cardiac Imaging Modalities Among Prevalent Cases of Heart Failure According to the Place of Living, 2002-2016

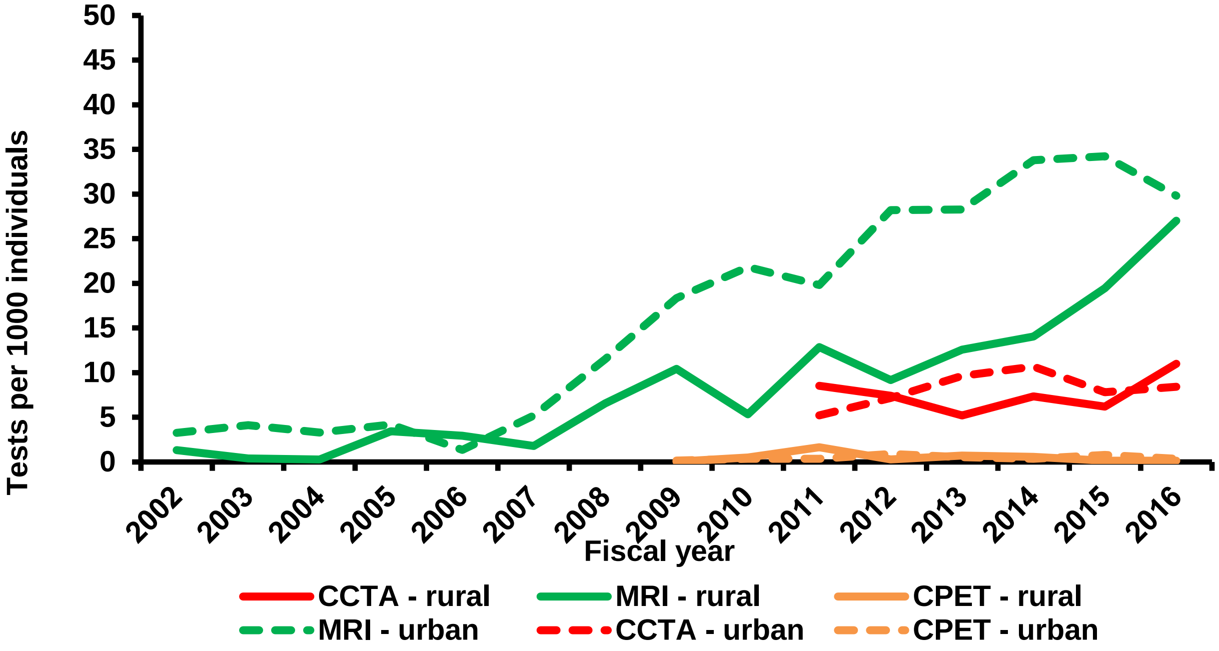

© 2019 Braga JR et al. *JAMA Network Open.*
